# Effects of Virtual Reality-Based Exercise Imagery on Pain in Healthy Individuals

**DOI:** 10.1155/2019/5021914

**Published:** 2019-04-17

**Authors:** Kazuhiro Hayashi, Shuichi Aono, Yukiko Shiro, Takahiro Ushida

**Affiliations:** ^1^Multidisciplinary Pain Center, Aichi Medical University, Nagakute, Japan; ^2^Department of Rehabilitation, Aichi Medical University Hospital, Nagakute, Japan; ^3^Department of Pain Data Management, Aichi Medical University, Nagakute, Japan; ^4^Department of Physical Therapy, Faculty of Rehabilitation Sciences, Nagoya Gakuin University, Nagoya, Japan; ^5^Institute of Physical Fitness, Sports Medicine, and Rehabilitation, Aichi Medical University, Nagakute, Japan

## Abstract

**Objective:**

Virtual reality (VR) is an advanced technology that can be used to attenuate pain. The present study aimed to investigate which method was more effective for pain management: VR combined with exercise imagery or VR distraction.

**Methods:**

Fifty-two healthy students participated in this randomized cross-over controlled trial. One VR-based task aimed to passively use the imagery of driving a car as a distraction intervention (the driving group), whereas the other VR-based task aimed to use exercise imagery (running) to actively engage the participants in movement (the running group). The mechanical pressure pain thresholds of the quadriceps and forearm and the heat pain threshold of the hand of each subject were measured before, during, and after each VR task. The differences between the values at each time point and the differences between the groups were analyzed.

**Results:**

The pressure and heat pain thresholds were significantly greater during VR task than those before VR task in both driving and running groups. The changes in the pressure pain thresholds that occurred during VR task were significantly higher in the running group than in the driving group. The difference between groups gradually declined after VR task. Conversely, there was no significant difference in the changes in the heat pain thresholds between the groups both during VR task and after VR task.

**Conclusions:**

VR combined with exercise imagery has a greater effect on pressure pain thresholds, but not heat pain thresholds, than VR distraction.

## 1. Introduction

Virtual Reality (VR) has been used in various medical applications during the past two decades. VR presents the participant with a 360° illusion of being completely surrounded by a virtual environment via a head-mounted display that tracks the motion of the participant's head. According to several reviews conducted in the field of pain management [1–3], many studies have evaluated VR as a means to attenuate pain perception. The effects of VR on pain have been revealed through a variety of mechanical and thermal modalities [3]. It has been reported that the primary mechanism through which pain perception is attenuated is via distraction, although other nondistraction mechanisms have been proposed [3–6]. VR is effective at reducing pain independent of the participant's imagery ability; however, imagery without VR is effective with only participants with high imagery abilities [7]. The effects of VR on pain are influenced by the sense of presence in the VR environment and various psychological factors [6]. The VR methodologies commonly used for pain management include playing video games [8] and watching video clips [9]. Watching video clips in VR is often combined with enhancement of pain control [10], cognitive load [11], hypnosis [12], and exercise imagery [13]. However, few experimental studies have investigated the different VR methodologies for pain management [10, 11, 14–16]. It was previously reported that the passive behavior of watching video clips had effects on pain than interactive behavior, although both were effective [14–16]. Meanwhile, Loreto-Quijada et al. suggested that there is no significant difference in the effects on pain between VR combined with enhancement of pain control and VR distraction [10]. Furthermore, Demeter et al. reported that there was no significant difference in pain reduction between high and low cognitive load environment interventions [11]. Although VR combined with a cognitive task demonstrated no superiority for pain management, the effects of other combinations on pain have not yet been investigated.

Exercise imagery intervention, which has been recently proposed as a treatment for pain [13, 17–19], is the mental representation of an action without any concomitant body movement [20]. The brain regions that are active during exercise imagery highly overlap with those responsible for actual movement, but exercise imagery results in low levels of activation [21, 22]. Based on this evidence, several studies have promoted exercise imagery as a therapeutic tool. Clinically, exercise imagery has been shown to attenuate pain in patients following total knee arthroplasty [17] and in those with shoulder impingement syndrome [18], chronic shoulder pain [19], and nonspecific lower back pain [13].* Participants imagine the same movement* as seen just prior to the exercise imagery [17–19] or while watching a video clip of the movements [13]. Although the precise mechanisms of exercise imagery intervention for pain management are still unclear, it is believed to occur via both pain distraction and punctually activated brain regions involved in the pain matrix network [17, 18]. VR combined with exercise imagery could potentially have a superior effect on pain management compared with VR distraction; however, this has yet to be demonstrated. The video clip except for exercise is eligible for VR distraction, because exercise observation activates the same neural regions as those involved in the actual execution of the observed action [23].

In the present study, we hypothesized that VR combined with exercise imagery would have a greater effect on pain than VR distraction, regardless of exercise imagery ability. Thus, the present study aimed to investigate which method was more effective for pain management: VR combined with exercise imagery or VR distraction.

## 2. Methods

### 2.1. Participants

We recruited healthy subjects to participate in the present study by means of posting flyers on a notice board. The inclusion criteria for participation were as follows: (1) between 20 and 24 years of age and (2) no ongoing pain issues. The exclusion criteria included a history of chronic pain conditions or serious health conditions and use of analgesics or other medications.

The sample size was calculated using G∗Power software (v3.1.9.2, Franz Faul, Universität Kiel, Kiel, Germany). Based on an effect size of 0.4 for the pain threshold [10, 11], the minimum number of subjects required was estimated to be 52 for an *α*-level of 0.05 and a power (1-*β*) of 0.80.

This study was approved by the Ethics Committee of Aichi Medical University. All subjects provided informed consent prior to participation in this study.

### 2.2. Experimental Design

**Figure 1:**
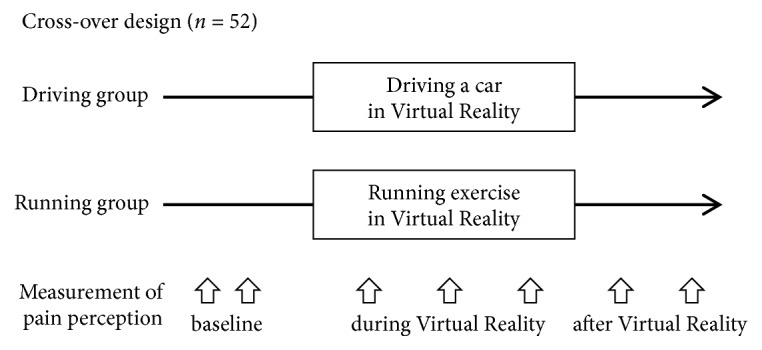
Experimental design.

Fifty-two subjects were included in the study. A flow diagram depicting the experimental design is presented in Figure 1. A randomized cross-over design was used to investigate the difference in the effects on pain between exercise imagery (the running group) and distraction in VR (the driving group). The order of VR methodologies was randomly determined using a random number table. A set of pain perception measurements were performed before, during (three times, at two-minute intervals), and 2 and 4 minutes after participating in each VR task.

The participants visited the laboratory on two occasions, with each visit occurring on a separate day. During the first visit, a psychological questionnaire using the State-Trait Anxiety Inventory Questionnaire (STAI) was administered and each subject's exercise imagery ability was measured prior to VR task.

### 2.3. Experimental Conditions

The experiment was performed in a university laboratory, where the room temperature was maintained at 26°C. Subjects remained seated in a fixed chair, with their backs supported by the backrest and their hips and knees positioned at 90°. The subjects placed their dominant forearm on a desk. The VR tasks and pain perception measurements were performed in this position.

### 2.4. VR Task

The participants viewed the virtual environment through a head-mounted display that provided a fully immersive experience [13]. The head-mounted display used was an HTC Vive (HTC Corporation, Taoyuan, Taiwan), with a resolution of 2160 × 1200 pixels, a latency of 22 milliseconds, and a refresh rate of 90 Hz, which produced less latency than its predecessors. The Vive Lighthouse system is comprised of two beacons placed on opposite ends of the room that emit infrared light via LEDs. These beacons, or base stations, track physical head orientation in the head-mounted display and present the virtual world accordingly.

**Figure 2:**
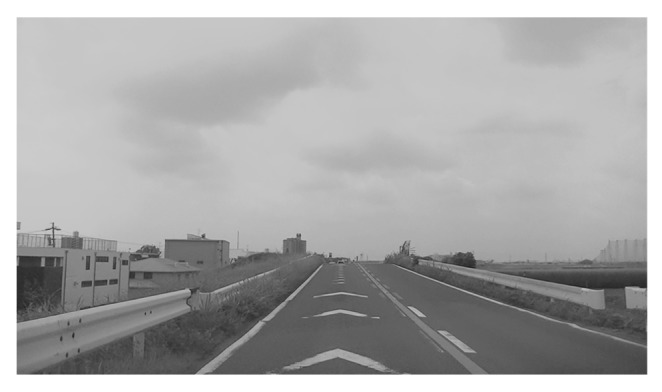
Schema illustrating the method of virtual reality.

The video clip was modeled with QBiC PANORAMA (Elmo, Nagoya, Japan) and Video Stitch Studio (Orah, Paris, France). The VR video footage was obtained by a cameraman who moves on the road, in a first-person view (Figure 2).

In the driving group, the participants watched the video clip and were asked to imagine themselves moving a car; this served as the distraction intervention. In the running group, participants watched the video clip and were asked to imagine themselves performing the running activity as vividly as possible while watching the video clip [13, 17, 18].

Before conducting the exercise imagery, the experimenter read an imagery script to ensure that all participants received similar exercise imagery instructions [17, 18]. Following the VR task, all participants were asked the following question: “To what extent did you feel like you were inside the virtual world during the VR task?” [24].

### 2.5. Measurement of Pain Perception

Pain perception was measured using pressure pain thresholds (PPT) and heat pain thresholds (HPT) [25]. We used the pain thresholds test because steady supra-threshold test stimuli could result in a floor effect due to its relatively low initial painfulness [26]. The participants remained seated in a fixed chair with their hips and knees positioned at 90°. The mechanical force transmitted to the muscle was measured using a calibrated mechanical pressure algometer (Digital Force Gage, AIKOH, Osaka, Japan). The rubber tip of the algometer was 1 cm in diameter. The PPT were measured at two sites: the left quadriceps muscle (half the distance between the anterior superior iliac crest and the superior patella) and the right dorsal forearm (half the distance between the lateral epicondyle of the humerus and the styloid process of the radius). The algometer was applied to the testing site, and the pressure was gradually increased approximately 5 N/s during the test. Participants were instructed to respond immediately when they felt pain from the pressure applied, at which point pressure testing ceased and the results were automatically recorded. All mechanical stimuli were applied by a single examiner who had partaken in extensive practice using an electric balance to ensure that the algometer was successfully applied to the participants in a consistent manner.

The HPT were assessed using a computer-controlled surface thermode (Intercross-200, Intercross Co., Tokyo, Japan) covering a 25 × 25 millimeter skin area on the palm of the left hand. The ascending method of limits was used, wherein the temperature stimulus was initiated at a baseline of 32°C and then was increased 1.0°C/second to a maximum of 50°C. The study participants were instructed to press a handheld switch as soon as they detected the sensation of heat pain, which was defined as the first sensation of pain. The peak temperature was stored and the thermode instantly decreased its temperature (3.0°C/second) back to baseline (32°C).

### 2.6. Measurement of Exercise Imagery Ability

#### 2.6.1. Delta Time between the Timed Up and Go Test (TUG) and the Imagined TUG

Mental chronometry, which is the measurement of the difference between the actual and imagined durations of movement execution, was used to assess each participant's exercise imagery ability using the delta time between the TUG and Imagined TUG [27, 28]. All participants completed one trial, first performing the TUG, followed by the Imagined TUG while sitting in a chair. The participants were instructed to seat, use the armrests to stand up,* walk 3 meters*, turn around, then walk back to the chair, and sit down. A stopwatch was started on the command of “Ready, set, go” and was stopped as the subject sat down at their self-selected normal speed. For the imagined test, the subjects were seated and were instructed to imagine performing the TUG test (*i.e.*, the Imagined TUG) and to say “Stop” aloud upon completion. Subjects could choose whether to do the Imagined TUG with their eyes open or closed. A stopwatch was started on the command of “Ready, set, go” and was stopped when the subject voiced the word “Stop.” The times for each test were recorded using a stopwatch to the nearest 0.01 second. A smaller time difference represented better imagery ability.

The following formula was used to calculate delta time between the TUG and Imagined TUG:

(1) TUG−Imagined  TUG/TUG+imagined  TUG/2×100

#### 2.6.2. The Movement Imagery Questionnaire-Revised (MIQ-R)

All participants completed the MIQ-R to evaluate their individual abilities to form kinesthetic and visual mental images [29, 30]. The MIQ-R is an eight-item self-report questionnaire that assesses the movement imagery ability for four basic movements: a knee lift, jump, arm movement, and waist bend. The ease of imaging is measured in both visual and kinesthetic modalities. For each movement, the participants first read a description and then physically performed the described movement. They subsequently assumed the same starting position and either visually or kinesthetically imagined performing the movement. Following these steps, participants rated their ease of imagery on a 7-point Likert-type scale (from 1 = “Very hard to see/feel” to 7 = “Very easy to see/feel”). The items for each subscale were averaged; a higher score represented better imagery ability.

### 2.7. Psychological Questionnaire

Each participant's anxiety status was measured using the STAI; items for state anxiety were selected for their ability to discriminate between stress and nonstress conditions [31, 32]. The state anxiety and trait anxiety subscales each consist of 20 statements that are designed to contain anxiety-present and anxiety-absent factors; all items are rated on a 4-point scale. To obtain subtest total scores, the 20 individual response scores for each subscale are summed; the total score for each ranges from 20 to 80, with a lower score reflecting a better anxiety status.

### 2.8. Statistical Analysis

The data were first normalized to baseline values. The percent change between the pre- and post-VR pain perception values was calculated [(post – pre) / pre × 100 [%]] separately for each pain perception. We initially tested if the data were normally distributed using the Shapiro-Wilk normality test. Since there were no normally distributed variables, nonparametric tests were performed for all analyses. Friedman's repeated-measures analysis of variance by ranks was used to calculate the temporal changes in outcome measures within each group. When a significant time effect was observed, Steel's method for pairwise multiple comparisons was applied to discriminate a significant difference from the baseline value. The statistical differences between the study groups were analyzed using the Wilcoxon signed-rank test. The correlations of variables were analyzed using Spearman's rank correlation coefficient. All graphs plot the mean ± the standard error of the mean (SEM), unless noted otherwise. JMP software (version 13, SAS Institute, Cary, NC, USA) was used for the calculations. A P value of <0.05 was considered statistically significant.

## 3. Results

### 3.1. Participant Characteristics

Table 1 presents the participants' demographics, pain perception values, exercise imagery abilities, and psychological factors. All subjects completed the study protocol. Regarding exercise imagery ability, the mean delta time between the TUG and Imagined TUG was 7.8, the mean MIQ-R visual subscale score was 23.4, and the mean MIQ-R kinesthetic subscale score was 23.3. All participants responded that they experienced a strong sense of entering or of being completely inside the virtual world.

### 3.2. PPT of the Quadriceps and Forearm

**Figure 3:**
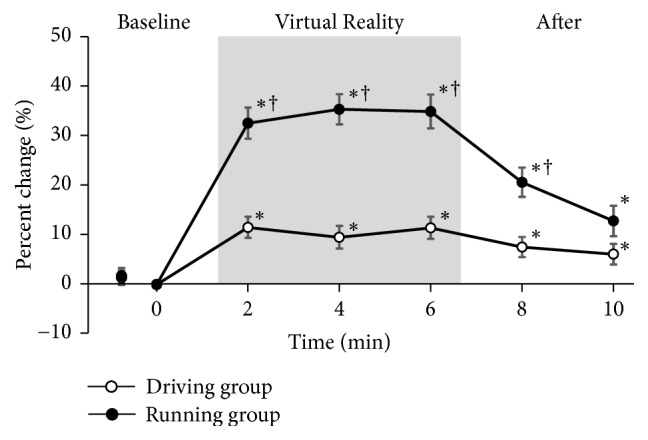
*Percent change for the PPT of the quadriceps*. PPT, pressure pain thresholds. Values are normalized to baseline values and expressed as mean ± SEM. ∗: p < 0.05 vs. baseline; †: p < 0.05 vs. driving group. Significance level is less than 5%.

**Figure 4:**
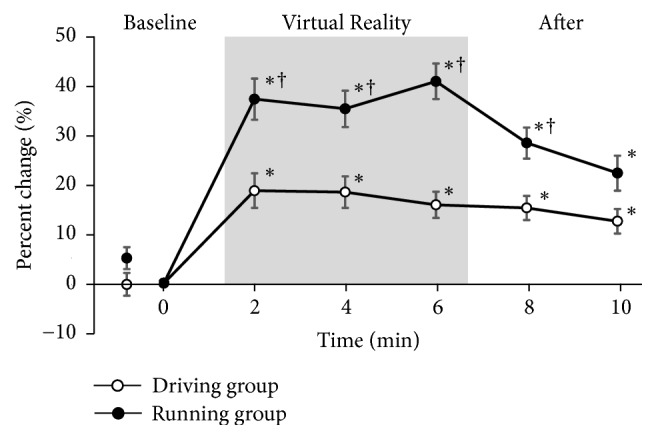
*Percent change for the PPT of the forearm*. PPT, pressure pain thresholds. Values are normalized to baseline values and expressed as mean ± SEM. ∗: p < 0.05 vs. baseline; †: p < 0.05 vs. driving group. Significance level is less than 5%.

Figures 3 and 4 depict the percent change for the PPT of the quadriceps and forearm, respectively.

In the driving group, the PPT of the quadriceps and forearm were significantly greater (>10%) than the baseline values. These threshold increases were maintained for >4 minutes after VR task.

In the running group, the PPT of the quadriceps and forearm were significantly greater (>30%) than the baseline values. The threshold was significantly higher in the running group than in the driving group. The threshold in the running group gradually returned to the baseline levels; there was no significant difference between the running and driving groups 4 minutes after VR task.

### 3.3. HPT of the Hand

**Figure 5:**
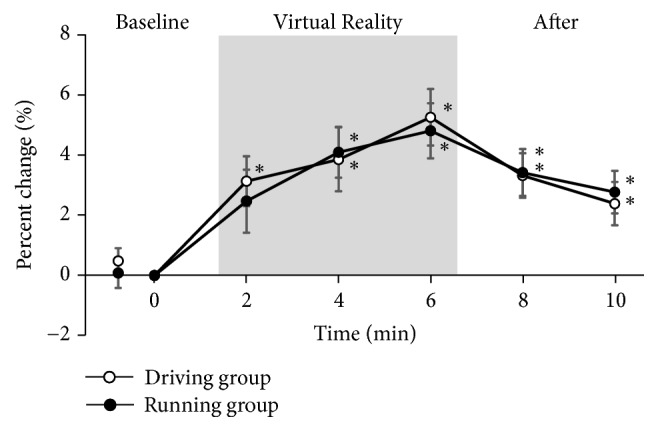
*Percent change for the HPT of the hand*. HPT, heat pain thresholds. Values are normalized to baseline values and expressed as mean ± SEM. ∗: p < 0.05 vs. baseline. Significance level is less than 5%.

The percent change for the HPT is presented in Figure 5. The HPT significantly increased from the baseline values in both driving group and running group. Both groups' thresholds significantly gradually increased during VR task and then decreased from the baseline values after VR task. Both groups' threshold increases were maintained significantly for >4 minutes after VR task. There were no significant differences in the HPT between the groups throughout the study protocol.

### 3.4. Correlations of Pain Perception with Exercise Imagery Ability and Psychological Factor Scores

Table 2 presents the correlations between the percent change of pain perception and the exercise imagery ability and psychological factor scores. The percent change for the PPT and HPT for each VR task showed no significant association with the delta time between the TUG and Imagined TUG, MIQ-R score, or STAI status. The difference in the percent change between the groups had no significant association.

## 4. Discussion

The present study investigated which method is more effective for pain management, VR combined with exercise imagery or VR distraction. The major findings of this study were as follows: (1) VR combined with exercise imagery had a greater effect on the PPT, but not the HPT, than VR distraction; and (2) the effects of VR combined with exercise imagery on pain occurred regardless of the participants' exercise imagery abilities or anxiety status.

Exercise imagery increases motor and premotor cortex activity, similar to what occurs during actual body movements [21, 22]. Motor cortex activity induced by repetitive transcranial magnetic stimulation without actual movement has been reported to have an analgesic effect [33]. Furthermore, other previous reports have demonstrated that exercise imagery attenuates pain [13, 17–19], which is consistent with the findings of the present study. The differences in the effects of exercise imagery according to specific pain modalities and/or body parts have not yet been investigated, but actual execution attenuates pain perception in multisegmental manifestations [34]. The present study utilized running imagery that included both lower and upper limb movements and thereby demonstrated the superior effects of VR combined with exercise imagery versus VR distraction on the PPT and that the effects were similar in both quadriceps and forearm.

VR-based interventions can serve as effective manipulations for pain reduction in individuals with efficient conditioned pain modulation, that is, an endogenous inhibitory pain system [35]. It has been previously demonstrated that having a conditioned pain modulation has a large effect on the PPT, but very little effect on the HPT [36, 37]. Similarly, in the present study, the HPT during VR task exhibited only slight increases (approximately 5%) compared to those that occurred in the PPT (>10%). Moreover, VR combined with exercise imagery had no superior effect on HPT compared with VR distraction. A systematic review demonstrated that actual aerobic exercise has a moderate effect size for the PPT (effect size = 0.58), but that for the HPT is slight (effect size = 0.04) [38]. These differences regarding the pain modalities are considered to be explained by the difference in brain activity that occurs with different pain stimuli [38]. However, further research is required to confirm the difference in the effects for different pain modalities [37, 38].

A participant's psychological factors, such as anxiety, will influence the effects of VR on pain [6]. However, no association was observed between the changes in pain and the participants' STAI statuses in the present study. Our study included young healthy individuals; thus participants often looked forward to performing the VR tasks without experiencing an extreme worsening of their anxiety status. The effects of imagery without VR differ according to the participant's imagery ability [7]. Conversely, VR-based imagery changes pain perception regardless of the participant's imagery ability [7]; this was also observed in the present study. However, it should be noted that there were no participants with extremely low exercise imagery abilities included in the present study, thus guaranteeing homogeneity enough in terms of individual ability to elicit exercise imagery. Further investigation of the difference of the association in healthy individuals and patients with clinical acute and chronic pain conditions is required.

The mechanisms that include VR effects pain are primarily thought to occur via pain distraction and by reducing pain-related brain activity in the anterior cingulate cortex, primary and secondary somatosensory cortex, insula, and thalamus [39, 40]. However, VR remains somewhat enigmatic with regard to its underlying neurobiological mechanisms. Theories beyond simple distraction have been proposed to explain the effects of VR [3–6]. For example, it has been postulated that its effects originate from intercortical modulation among signaling pathways of the pain matrix through attention, emotion, memory, and other senses, thereby producing analgesia. A VR environment is capable of manipulating an even more complex set of cognitive and emotional conditions than the presentation of the most classic cognitive tasks.

Many studies have used VR to attenuate pain among individuals with burn injuries, complex regional pain syndrome (CRPS), phantom limb pain, chronic pain, and during diagnostic and therapeutic procedures [1–3]. To date, VR for pain management has most often utilized as “VR for distraction” or “VR for actual execution of pain-related movement patterns” methods [1]. Use of VR with movement of the affected regions has been shown to have a larger effect on the attenuation of pain perception than with use of only passive VR distraction [41]. The present study showed that VR for exercise imagery had a greater effect on the pressure pain thresholds than VR distraction, although it was not compared with VR with movement such as playing video games [8]. VR for exercise imagery potentially provides a means to expose patients to movements that they may otherwise avoid due to pain or fear. The competitive methodologies among VR for distraction, VR for exercise imagery, and VR for actual execution of pain-related movement patterns should be investigated in clinical patients.

## 5. Study Limitations

The present study had several limitations. First, the exercise imagery used only included running. Although it has been demonstrated that the most types of actual exercise attenuate pain perception, the largest effect sizes have been observed following isometric exercise [38]. Furthermore, actual exercise attenuates pain perception more in the moving body parts than in the nonmoving body parts [34]. Suitable exercise imagery methodologies should be elucidated for specific diseases. Second, the effects of exercise imagery without VR on pain were not investigated. Further studies are required to clarify the effects of VR combined with exercise imagery on pain. Third, the present study assessed subjective pain perception only at limited points with limited modalities. Further evaluation should be conducted using different modalities in multiple areas of the body and objective measurement, such as brain activity, in future studies. Fourth, the present study did not assess the participants' psychological factors [10], autonomic nervous systems, supra-threshold pain stimuli, pressure and heat perceptions, or raw values of pain thresholds. Fifth, VR has an issue of cybersickness, the bodily discomfort associated with exposure to VR task [42]. Display factors and task performance could be instrumental in reversing cybersickness [42]. Finally, this study investigated only the immediate effects in healthy individuals. Therefore, the generalizability of these results among subjects with chronic pain conditions is even absent.

## 6. Conclusions

VR combined with exercise imagery had a greater effect on the PPT, but not the HPT, than VR distraction. The effects of VR combined with exercise imagery on pain occurred regardless of the participant's exercise imagery ability or anxiety status.

## Figures and Tables

**Table 1:** Participant's characteristics.

*Demographics*	
Male gender, n (%)	37 (71%)
Age (years)	21 (0.9)
*Pain perception*	
PPT of the quadriceps (N)	61.8 (20.0)
PPT of the forearm (N)	34.7 (11.7)
HPT of the hand (°C)	42.5 (3.2)
*Exercise imagery ability*	
MIQ-R visual subscale (points)	23.4 (3.2)
MIQ-R kinesthetic subscales (points)	23.3 (3.2)
Delta time between TUG and imagined TUG (%)	7.8 (4.7)
(i) Imagined TUG (seconds)	5.9 (1.6)
(ii) Performed TUG (seconds)	7.7 (1.2)
*Psychological factors*	
STAI state subscale (points)	23.4 (4.8)
STAI trait subscale (points)	29.6 (4.3)

PPT, pressure pain thresholds; HPT, heat pain thresholds; MIQ-R, Movement Imagery Questionnaire Revised; TUG, Timed Up and Go test; STAI, State-Trait Anxiety Inventory.

Data from continuous variables are shown in mean and standard deviation (SD). Data from categorical variables are shown in number and (%) of patients.

**Table 2:** Correlation coefficients between changes in pain and variables.

	Driving group			Running group			Difference changes between groups		
	Percent change for PPT of the quadriceps	Percent change for PPT of the forearm	Percent change for HPT of the hand	Percent change for PPT of the quadriceps	Percent change for PPT of the forearm	Percent change for HPT of the hand	Percent change for PPT of the quadriceps	Percent change for PPT of the forearm	Percent change for HPT of the hand

*Exercise imagery ability*									
MIQ-R visual subscale	-0.050	0.058	0.049	0.155	0.187	0.242	0.090	0.054	0.107
MIQ-R kinesthetic	-0.098	0.041	0.103	0.072	0.204	0.209	0.085	0.120	0.090
subscales									
Delta time between TUG	0.158	0.085	0.038	0.226	0.212	0.181	0.154	0.233	0.166
and imagined TUG									
*Psychological factors*									
STAI state subscale	-0.068	-0.021	-0.157	-0.248	-0.044	0.005	-0.210	-0.069	0.170
STAI trait subscale	-0.189	-0.054	-0.031	-0.063	0.008	0.014	0.005	-0.034	0.093

PPT, pressure pain thresholds; HPT, heat pain thresholds; MIQ-R, Movement Imagery Questionnaire Revised; TUG, Timed Up and Go test; STAI, State-Trait Anxiety Inventory.

The values of Spearman's correlation coefficients are shown. There was no significant correlation between changes in pain and variables (p > 0.05).

## Data Availability

The data used to support the findings of this study are available from the corresponding author upon request.
